# Giant Sequoia (*Sequoiadendron giganteum*) Foliar Distillate Elicits Sex-Biased Attraction by the Western Cedar Bark Beetle (*Phloeosinus punctatus*) in Laboratory Assays

**DOI:** 10.1007/s10886-026-01775-7

**Published:** 2026-11-14

**Authors:** Gabriel G. Foote, Nathaniel E. Foote, Thomas S. Davis

**Affiliations:** 1 Forest & Rangeland Stewardship, Warner College of Natural Resources, Colorado State University, Fort Collins, CO USA; 2 Department of Entomology and Nematology, College of Agricultural and Environmental Sciences, University of California, Davis, Davis, CA USA

**Keywords:** *Phloeosinus*, *Sequoiadendron*, Olfactometry, Kairomones

## Abstract

Giant sequoia (*Sequoiadendron giganteum*) is a globally recognized cultural resource and recreational attraction that is listed as ‘endangered’ on the International Union for Conservation of Nature (IUCN) Red List. In recent years, the western cedar bark beetle, *Phloeosinus punctatus*, has been implicated as a contributing factor in giant sequoia crown dieback and mortality of mature trees weakened by drought stress and severe wildfire. However, semiochemical options for monitoring and potentially managing *P. punctatus* populations are presently unavailable. To address this, we evaluated orientation behavior of *P. punctatus* to a steam-extracted distillate prepared from giant sequoia branches using Y-tube olfactometry. During olfactometry assays, 45% of adult beetles chose the distillate while 22.5% oriented towards the control arm (32.5% made no choice). Multinomial contrasts indicated that females oriented toward the distillate, whereas males exhibited no preference. Results from gas chromatography–mass spectrometry (GC–MS) found the volatile headspace of the distillate to be primarily (> 90%) composed of monoterpenes along with lesser proportions of oxygenated monoterpenoids and relatively non-volatile phenylpropanoids (< 10%), several of which are documented kairomones for conifer-associated insects. Likely kairomones for *P. punctatus*, the role of females as the host-seeking, pioneering sex, and the potential for giant sequoia distillate and synthetic terpene standards to support monitoring across the natural range of the host tree are discussed.

## Introduction

Giant sequoia (*Sequoiadendron giganteum* (Lindl.) J. Buchholz; Cupressales: Cupressaceae) is among the largest and longest-lived terrestrial organisms on Earth, with individuals capable of exceeding 60 m in height and living for more than three millennia (Hartesveldt et al. 1975; Flint 1987; Stephenson 2000; Sillett et al. 2015). The natural distribution of giant sequoia is restricted to ~ 70 groves totaling roughly 11,000 ha on the western slope of California’s Sierra Nevada mountains, predominantly on federally managed lands (Rundel 1972; Weatherspoon 1990). Giant sequoia is a globally recognized cultural resource and recreational attraction and is further listed as ‘endangered’ on the International Union for Conservation of Nature (IUCN) Red List, with fewer than 80,000 mature trees remaining in its natural range (Dilsaver and Strong 1990; Schmid and Farjon 2013). Critically, several instances of foliar dieback in the crown and subsequent mortality of giant sequoia associated with hotter droughts and high-severity fires have been reported in the Sierra Nevada in recent years (Nydick et al. 2018; Stephenson et al. 2018; Stephenson and Brigham 2021; Shive et al. 2022; Soderberg et al. 2024).

Breakage of large branches, upper-crown material, and, infrequently, entire trees is common in mature giant sequoias during strong winds and winter storms (Foote et al. 2024; Radecki and Davis 2026). This accumulation of severed branch material on the ground is optimal breeding substrate for *Phloeosinus punctatus* LeConte (Coleoptera: Curculionidae: Scolytinae), the primary bark beetle associate of giant sequoia. This beetle species overwinters in giant sequoia crowns and begins locating host material on the ground during early spring (Foote et al. 2024). This behavior is likely governed by temperature accumulation (Damos and Savopoulou-Soultani 2012; Foote et al. 2024), with warmer conditions potentially altering the onset and duration of flight activity in *P. punctatus*, consistent with temperature-dependent phenological responses documented in other scolytine species inhabiting temperate coniferous forests (Gaylord et al. 2008; Dell and Davis 2019). This species is monogamous and is estimated to yield ~ 30 progeny per kilogram of branch material under native-range field conditions (Foote et al. 2024). The beetle is presumed to reproduce at equal sex ratios (Burke 1966). However, the respective roles of females and males in long-range host location and mate attraction remain unresolved. Their development proceeds through four larval instars over approximately 6–8 weeks, after which adults are presumed to return to giant sequoia crowns and construct shallow, short (typically less than 1 cm) excavations, or ‘hibernacula,’ in live branches before entering quiescence (DeLeon 1952; Burke 1966; Foote et al. 2024). It is unknown whether the construction of these hibernacula has an impact on host tree vigor, especially when occurring at relatively high abundance or frequency.

While previously considered non-pestiferous towards their host, a growing number of recent observations of *P. punctatus* presence in giant sequoia crowns has increased concern among regional land stewards and ecologists regarding whether this species is a contributing factor in crown dieback and mortality of mature trees weakened by drought and fire-related stress (Stephenson et al. 2018; Shive et al. 2022; Foote et al. 2024; Radecki and Davis 2026). Notably, there are recent field observations of *P. punctatus* reproducing in the aerial branches of dying or severely stressed hosts (A. Ambrose and W. Baxter pers. comm., August 2025). However, semiochemical lures for trap-based population monitoring or management of *P. punctatus* in giant sequoia are presently unavailable. More broadly, general knowledge of semiochemically mediated giant sequoia–arthropod interactions is lacking.

A limited number of studies have characterized giant sequoia volatile oils and resins from cultivated trees outside the species’ native range. In Oregon, USA, α-pinene—the primary constituent of giant sequoia foliage oil and a widespread, frequently abundant monoterpene in conifer foliage and resin (Malhocká and Švábová 2023)—declined as the growing season progressed, whereas safrole, eugenol, methyl eugenol, and elemicin—volatile aromatic compounds belonging to the phenylpropanoid class—accumulated as new foliage matured (Levinson et al. 1971). Similarly, needle and wood oils from trees cultivated in Croatia were composed largely of α-pinene (approximately 45% in each tissue on average), although oil yields (0.25% vs. 0.014% w/w) and secondary composition differed between tissues (Jerković et al. 2003). Resin composition has also been documented to vary among giant sequoia trunk, needle, and female-cone tissues in ornamentally planted trees in the Czech Republic, with a relatively nonvolatile podocarpane-type diterpenoid—a member of a compound class broadly associated with conifer defense against herbivorous insects and fungal pathogens—accounting for roughly 20%, one-third, and 60% of resin in these tissues, respectively, and nearly twice the abundance of α-pinene in cones (Malhocká and Švábová 2023).

Several of the monoterpenes documented in these phytochemical studies of giant sequoia—including α-pinene, β-pinene, camphene, myrcene, limonene, and Δ-3-carene—have been implicated in scolytine host location, either as attractants or as modifiers of pheromone-mediated responses (Chénier and Philogène 1989; Byers et al. 1990; Seybold et al. 2018). Therefore, extracts of giant sequoia branch material—or synthetic formulations based on key constituents of its volatile profile—may have applications as lures for ecological research and population monitoring of *P. punctatus*.

Steam distillation—wherein host plant phytochemicals are extracted and subsequently characterized (e.g., via gas chromatography–mass spectrometry [GC–MS])—is a common initial step in the development of semiochemical tools for arthropod management (Metcalf and Kogan 1987; Akdağ and Öztürk 2019; Barbosa-Cornelio et al. 2019; Tavares et al. 2022). Behavioral (e.g., olfactometry) and physiological (e.g., electroantennographic detection [EAD]) assays are then commonly conducted to determine which compound(s) elicit attraction or deterrence in target taxa (Wood et al. 1966; Byers et al. 1990; Miller 2006; Miller and Rabaglia 2009; Brezolin et al. 2018; Seybold et al. 2018). Behaviorally relevant compounds and blends can then be prioritized during field testing (Zhang et al. 2008; Bozsik et al. 2016, 2022). Principal research relevant to the chemical ecology of *P. punctatus* includes work on *Phloeosinus aubei* Perris in Europe, where host-tree phytochemical extracts have been used to identify kairomones and develop lures (Bozsik and Szőcs 2017; Fiala et al. 2024; Bozsik et al. 2025). Comparable research on *Phloeosinus serratus* LeConte attraction towards host tree extracts has also been conducted in juniper woodlands of western North America (Hayes et al. 2008).

Here, we assessed the attraction of *P. punctatus* towards a steam-extracted giant sequoia foliar distillate (hereafter, distillate) derived from fresh branch material using Y-tube olfactometer assays. We further characterized the phytochemical composition of this product and its dynamic headspace using GC–MS. We conclude with discussion regarding (i) sex-biased orientation of *P. punctatus* towards host tree distillate, (ii) likely host-produced kairomones for *P. punctatus* and other insect associates of giant sequoia, and (iii) the potential use of giant sequoia distillate as well as synthetic analogs of its phytochemical constituents for *P. punctatus* monitoring.

## Methods and Materials

### Tree Branch Collection and Steam Distillation

Giant sequoia branch material (4.2 kg of foliage-bearing twigs and stems [0.5–4.0 cm in diameter]) was collected from Giant Forest in Sequoia National Park, CA, USA (36.55882, -118.75463) in May 2025. Branch material was harvested from the lower canopy (< 3 m above the root collar), vacuum-sealed, and kept on ice until processing (< 24 h). Selected branch material was chosen based on accessibility (removed via pole saw), as well as relative proximity to where *P. punctatus* adults were collected for the behavioral assays described below.

Fresh foliage-bearing branches collected from living trees were used as a standardized, terpene-rich source for distillate production and GC–MS characterization rather than naturally downed material of unknown post-detachment history. Essential-oil yield from giant sequoia needles is substantially greater than that from wood (0.25% vs. 0.014% w/w; Jerković et al. 2003), and studies of other conifers indicate that monoterpene emissions from wounded or cut woody tissues are strongly time dependent, typically declining after an initial post-injury release (Loreto et al. 2000; Kivimäenpää et al. 2012). The volatile composition of naturally downed branches would therefore be expected to vary with time since detachment and subsequent exposure to temperature, desiccation, oxidation, microbial activity, and insect colonization. Using freshly collected foliage-bearing branches reduced these uncontrolled sources of variation.

Giant sequoia distillate (unseparated aqueous + oil phases) was produced using a stainless steel essential-oil distillation kit (Clawhammer Supply Inc.; Tacoma, WA, USA). Steam was generated by heating distilled water to a boil while maintaining an internal boiler pressure of no more than 35 kPa; once the water reached a boil, plant material was added and distilled for 2 h. The distillate was homogenized into a single composite batch via gentle inversion and stored in a chemical storage jar at 4 °C until use in behavioral assays and chemical analyses described below. Prior to each olfactometer trial set (10 total; see below), the composite distillate was re-homogenized by vortexer for 30 s and a 30-mL aliquot was subdivided into three 10-mL portions for (i) a Y-tube olfactometer trial set (four individual tests per set), (ii) liquid–liquid extraction for GC–MS characterization of the aliquot used in that trial set, and (iii) dynamic headspace sampling of volatiles emitted from a matched portion during the Y-tube assays.

### Chemical Analysis of Giant Sequoia Steam Distillate and Associated Headspace Sampling

To characterize the phytochemical composition of the individual distillate aliquots (*n* = 10) used in each olfactometer trial set, a 10 mL subsample of each aliquot was subjected to liquid–liquid extraction with 5 mL of high-purity n-hexane. The mixture was gently vortexed for 30 s, allowed to separate, and the upper organic phase was recovered via a glass syringe. A 1.5 mL portion of the hexane extract was then transferred to a GC–MS vial fitted with a PTFE-lined cap. For headspace analysis, an additional 10 mL subsample of the same aliquot was placed in an odor chamber as described above. Volatile emissions were then collected by pulling clean, charcoal-filtered air through the chamber and into a volatile collection trap (10 cm glass tube packed with 25 mg Porapak Q; Volatile Collection Trap LLC; Gainesville, FL, USA) at 0.2 L min⁻¹ for 30 min using an oil-free diaphragm pump (Fish Lab Inc.; Winston, GA, USA). Trapped volatiles were eluted from the Porapak Q with 5 mL of high-purity (> 99%) n-hexane (Sigma Aldrich, Inc., St. Louis, MO, USA; product # HX0293; lot # SP307264) into a 10 mL glass vial, gently vortexed for 30 s, and transferred to a 2 mL GC–MS vial for chemical analysis. All samples were stored at − 20 °C until GC–MS analysis.

Chemical analysis of the distillate’s phytochemical composition and volatile headspace was carried out on an Agilent 7890B gas chromatograph coupled with a 5977B quadrupole mass spectrometer. The GC was fitted with an HP-5ms fused-silica capillary column (30 m × 0.25 mm ID, 0.25 μm film thickness) with helium as the carrier gas (constant flow 1 mL/min). The injection volume was 1.0 µL of the hexane extract in split mode (10:1 ratio). The oven temperature program was 45 °C for 2 min, followed by ramps of 3 °C/min to 180 °C, 5 °C/min to 280 °C, and 10 °C/min to 310 °C. The MS was operated in electron impact (EI) mode at 70 eV, scanning m/z 40–400.

Tentative compound identification relied on comparison of measured mass spectra with GC–MS spectra from MassBank of North America (MoNA) and the National Institute of Standards and Technology (NIST) library, and comparison of experimental retention indices (RIs) with published RIs from documented phytochemical profiles of giant sequoia foliage and wood (Levinson et al. 1971; Jerković et al. 2003; Garcia et al. 2017; Malhocká and Švábová 2023) and the NIST Chemistry WebBook (Linstrom and Mallard 2024). n-Alkane standards (C7–C40) were analyzed under the same oven program described above to derive experimental RI for each peak. Analytical standards (Merck KGaA/MilliporeSigma: Terpene Mix A [#CRM40755]; Terpene Mix B [#CRM40937]) were analyzed under the same GC–MS conditions as samples and used to verify terpene identities. Relative abundances of detected compounds were calculated via integration. Compounds were included in the statistical analyses reported below if (i) their library match probability and match factor (on a 0–999 scale) were ≥ 80% and ≥ 850, respectively, and (ii) they were detected in ≥ 7 samples within each sample type, with distillate and distillate headspace evaluated separately.

### Y-Tube Olfactometer Bioassays

Adult *P. punctatus* were collected from a downed, naturally colonized giant sequoia branch (~ 1.5 m long, 10 cm in diameter on average) in Giant Forest, Sequoia National Park, CA (36.55492, − 118.753755) in May of 2025. The branch was divided into 25 cm long sections, each of which was individually stored within a ventilated rearing container (clear polypropylene boxes; 11 × 8 × 5 cm each) indoors at a University of California, Davis (UCD) facility (mean temperature: 21 °C; mean relative humidity: 47%) underneath a grow light (Feit Electric Company; Pico Rivera, CA; Item No.: GLP8FS/CLAMP/32 W/LED) operating at a 14:10 (light: dark) photoperiod. Individuals from this F1 generation began emerging in late June 2025 and were stored at 4 °C until utilized in biological assays within a week of initial storage.

Behavioral responses of *P. punctatus* to giant sequoia distillate were evaluated between 1200 and 1500 h at 21 °C and under a laminar-flow hood using a glass Y-tube olfactometer (Sigma Sciences LLC; Micanopy, FL, USA) at UCD. To eliminate visual cues, the olfactometer was enclosed in and suspended within a black 19 L bucket. Acid-washed, charcoal-filtered air was delivered into the Y-tube via two equal streams (flow rate = 0.2 L min⁻¹ each), which passed through separate 4 L glass odor chambers before entering the olfactometer arms via a 30 cm length of polytetrafluoroethylene (PTFE) tubing (9.5 mm OD). Odor chamber lids were modified with two 304 stainless steel fittings (OD = 1 cm) to allow intake of filtered air through a 15 cm segment of PTFE tubing (outer diameter = 1.2 cm) containing 10 g of activated charcoal. The treatment chamber contained filter paper (diameter = 7.5 cm) loaded with distillate (10 mL), while the control chamber contained filter paper loaded with an equal volume of deionized water.

Each olfactometer trial set consisted of four sequential beetle tests using a single 10-mL distillate portion. Individual beetles (*N* = 40) were randomly selected, sexed (23 females, 17 males), and introduced at the base of the Y-tube, where they were allowed up to 3 min to make a choice. A choice was recorded if a beetle moved ≥ 2 cm into one arm and remained there for ≥ 5 s. Reversal of choice after 5 s was not recorded. Beetles that failed to choose within 3 min were recorded as “no response.” After every trial set, the assignment of treatment and control odors to the two arms was altered to ensure that treatment odors were presented on each arm with equal frequency. Filter papers were also replaced and loaded with fresh distillate after every trial set, and glassware was cleaned with acetone.

## Statistical Analyses

### Chemical Analysis of Giant Sequoia Steam Distillate and Associated Headspace Sampling

Averages and standard errors of individual compounds found in giant sequoia distillate and distillate headspaces were reported alongside their respective retention times (RTs) and experimentally derived retention indices (RIs). Relative abundances of individual compounds were expressed as normalized relative percentages. Distillate and distillate-headspace measurements were compared compound by compound using two-sided paired t-tests (stats::t.test(paired = TRUE) in R. Because multiple compounds were tested, P values were adjusted across all tested compounds using the Benjamini–Hochberg false-discovery-rate procedure (stats::p.adjust(method = “BH”); Benjamini and Hochberg 1995); an FDR-adjusted *P* < 0.05 was considered significant.

Grouped Y-tube olfactometer outcomes for female *P. punctatus* were analyzed in relation to trial-set-matched headspace constituent relative abundances (%) under small-sample, multicollinear conditions using ridge-penalized binomial logistic regression (L2-regularized logistic regression). The response was the probability of distillate-arm choice conditional on a female responding; no-choice outcomes were not included. For each of ten trial sets, numbers of responding females choosing the distillate and control arms were linked to the complete relative composition of 18 constituents measured from the matched headspace portion. These profiles were used as proxies for relative variation in the blends emitted during the corresponding trials. Individual constituents were not presented or tested separately. Because multivariate volatile profiles were compositional, zeros were replaced multiplicatively with zCompositions::multRepl() before centered log-ratio transformation with compositions::clr() (Aitchison 1982; van den Boogaart and Tolosana-Delgado 2008; Palarea-Albaladejo and Martín-Fernández 2015). Positive coefficients represented increased odds of distillate choice; coefficient stability was summarized by nonparametric resampling of complete trial-set/profile units with model refitting (Friedman et al. 2010). Positive and negative coefficients were interpreted only as directional, within-batch associations between relative blend composition and female choice, not as causal effects or evidence of behavioral activity of individual compounds.

Differences in multivariate composition between distillate and headspace were visualized using principal coordinates analysis (PCoA) with vegan::vegdist(method = “bray”) and ape::pcoa() (Dixon 2003; Paradis and Schliep 2019). Prior to analysis, compound peak areas (% relative abundance) within each sample were compiled into a sample × compound matrix; compounds absent from a sample were treated as zeros. Proportions were square-root transformed to reduce the influence of highly dominant compounds while retaining zeros, and pairwise compositional dissimilarities were calculated using Bray–Curtis dissimilarity. Compound vectors were fitted to the PCoA scores using vegan::envfit() (Dixon 2003).

### Y-Tube Olfactometer Bioassays

We analyzed Y-tube choices using multinomial logistic regression with three nominal outcomes: distillate, control, and no choice. Overall and sex-specific pairwise outcome contrasts were estimated from intercept-only models fitted to the full sample and separately by sex. Effects are reported as relative risk ratios (RRRs; exponentiated coefficients) with Wald 95% confidence intervals. An RRR > 1 indicates that the numerator outcome occurred more frequently than the denominator outcome, whereas an RRR < 1 indicates the reverse; intervals including 1.0 indicate no difference between outcomes.

Responder-only distillate-versus-control choices were evaluated with two-sided exact binomial tests; the female-versus-male responder comparison used a two-sided Fisher’s exact test, with the sample odds ratio and its log-Wald 95% confidence interval reported descriptively. Multinomial models were fitted using nnet::multinom() (Venables and Ripley 2002), Wilson intervals for observed proportions were calculated using binom::binom.confint(methods = “wilson”) (Wilson 1927; Dorai-Raj 2022), and exact tests were performed using stats::binom.test() and stats::fisher.test() in R (R Core Team 2025).

## Results

### Chemical Analysis of Giant Sequoia Steam Distillate and Associated Headspace Sampling

The distillate and corresponding headspace yielded 21 and 18 tentatively identified phytochemical constituents, respectively, accounting for 98.4% and 98.9% of the relative composition (Table 1). Both phases were dominated by monoterpene hydrocarbons, with α-pinene as the principal constituent in both the distillate (43.7 ± 0.19%) and the headspace (60.3 ± 8.2%). The distillate additionally contained a substantial phenylpropanoid fraction, including elemicin (13.3 ± 0.11%) and methyl eugenol (4.5 ± 0.02%); other abundant constituents included myrcene (7.6 ± 0.08%), terpinen-4-ol (5.7 ± 0.03%), and limonene (4.5 ± 0.05%). In contrast, the headspace was further enriched in volatile monoterpene hydrocarbons, particularly myrcene (10.9 ± 1.9%) and limonene (8.9 ± 3.2%), in addition to α-pinene (Table 1).

**Table 1:** Tentative volatile composition (%) of *Sequoiadendron giganteum* steam distillate and matched headspace extracted from foliage-bearing branch material collected from Sequoia National Park, CA, USA. Different superscript letters within a row indicate significant differences in relative abundance between distillate and headspace based on paired t-tests with Benjamini–Hochberg FDR correction (adjusted *P* < 0.05)

Compound	RT (min)	RI	Pub. RI	Relative content (% ± SE)	
				distillate	headspace
α-Pinene	8.08	939	943^1^	43.7 ± 0.19^b^	60.3 ± 8.2^a^
Camphene	8.57	954	959^1^	1.4 ± 0.01^b^	2.3 ± 0.8^a^
Sabinene*	9.07	969	965^2^	2.4 ± 0.03^a^	2.3 ± 0.5^a^
β-Pinene	9.40	979	986^1^	2.4 ± 0.02^b^	3.8 ± 0.7^a^
Myrcene	9.79	991	995^1^	7.6 ± 0.08^b^	10.9 ± 1.9^a^
Δ-3-Carene	10.48	1011	1005^2^	1.8 ± 0.02^b^	2.9 ± 1.1^a^
p-Cymene*	10.96	1024	1011^2^	1.4 ± 0.01^a^	1.5 ± 0.6^a^
Limonene	11.21	1031	1037^1^	4.5 ± 0.05^b^	8.9 ± 3.2^a^
γ-Terpinene	12.33	1062	1047^2^	0.7 ± 0.01^a^	1.1 ± 0.3^a^
Terpinolene	13.21	1086	1079^2^	1.1 ± 0.01^a^	1.2 ± 0.7^a^
Linalool	13.62	1097	1102^1^	0.5 ± 0.01^a^	0.5 ± 0.1^a^
Pinocarveol*	15.08	1138	1122^2^	0.5 ± 0.01	–
Borneol*	16.09	1165	1149^2^	0.5 ± 0.01^a^	0.2 ± 0.05^b^
Terpinen-4-ol	16.52	1177	1162^2^	5.7 ± 0.03^a^	1.0 ± 0.36^b^
α-Terpineol	16.96	1189	1172^2^	3.6 ± 0.04^a^	0.5 ± 0.18^b^
Safrole*	20.35	1285	1265^2^	1.4 ± 0.02^a^	0.5 ± 0.20^b^
Eugenol*	22.76	1357	—	0.6 ± 0.01^a^	0.2 ± 0.05^b^
Methyl eugenol*	24.32	1403	1368^2^	4.5 ± 0.02^a^	0.6 ± 0.17^b^
Elemicin*	28.83	1550	1519^2^	13.3 ± 0.11^a^	0.2 ± 0.09^b^
9,10-Dehydro- isolongifolene*	29.14	1561	—	0.5 ± 0.01	–
Nerolidol*	29.25	1565	1548^2^	0.3 ± 0.005	–

*Note*. RI = linear retention index calculated from the present GC–MS data; Pub. RI = closest published RI used as supporting identification evidence. Sources: 1 Malhocká and Švábová (2023); 2 Garcia et al. (2017). *Tentative identification based on EI–MS library match and RI comparison; no authentic-standard co-injection. — = no suitable numeric Pub. RI from the cited sources; – = not detected

When summarized by chemical class, monoterpene hydrocarbons accounted for 67.0% of the distillate and 95.2% of the headspace, whereas oxygenated monoterpenes comprised 10.8% and 2.2%, respectively. Phenylpropanoids represented 19.8% of the distillate but only 1.5% of the headspace, while minor sesquiterpene/oxygenated sesquiterpene constituents (0.8% total) were detected only in the distillate. The headspace contained significantly higher relative proportions of α-pinene, camphene, β-pinene, myrcene, Δ-3-carene, and limonene. In contrast, the distillate was significantly enriched in borneol (0.5 ± 0.01% vs. 0.2 ± 0.05%), terpinen-4-ol (5.7 ± 0.03% vs. 1.0 ± 0.36%), α-terpineol (3.6 ± 0.04% vs. 0.5 ± 0.18%), safrole (1.4 ± 0.02% vs. 0.5 ± 0.20%), eugenol (0.6 ± 0.01% vs. 0.2 ± 0.05%), methyl eugenol (4.5 ± 0.02% vs. 0.6 ± 0.17%), and elemicin (13.3 ± 0.11% vs. 0.2 ± 0.09%) relative to the headspace. Sabinene, p-cymene, γ-terpinene, terpinolene, and linalool did not differ significantly between matrices, whereas pinocarveol, 9,10-dehydro-isolongifolene, and nerolidol were detected only in the distillate (Table 1).

**Fig. 1:**
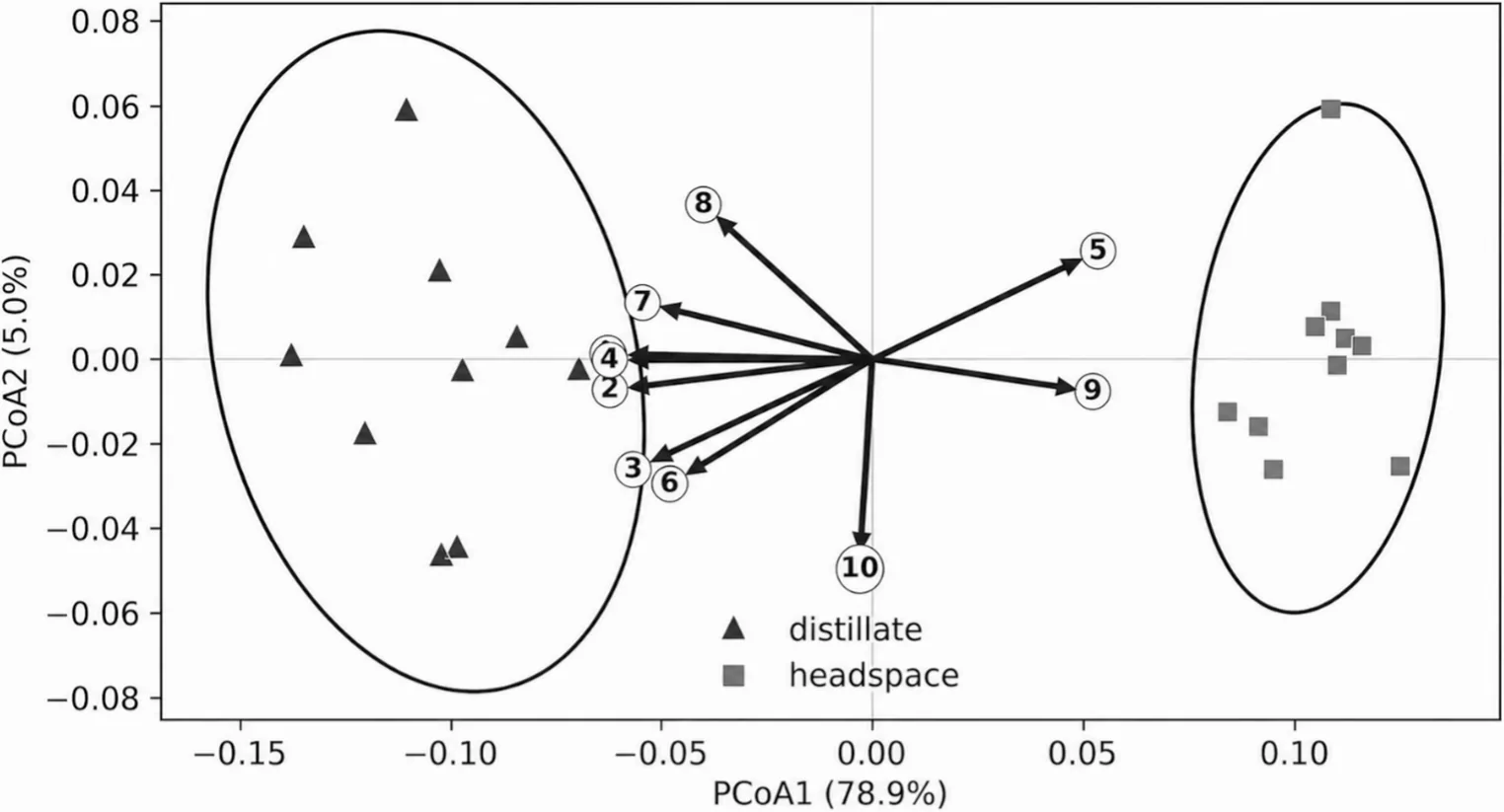
Bray–Curtis principal coordinates analysis (PCoA) of *Sequoiadendron giganteum* distillate and matched headspace chemical profiles based on square-root-transformed relative abundances. Triangles and squares distinguish distillate and headspace profiles, respectively; ellipses summarize the dispersion of the plotted analytical profiles. Numbered vectors show the ten compounds most strongly associated with the ordination configuration: (1) Terpinen-4-ol; (2) Elemicin; (3) α-terpineol; (4) Methyl eugenol; (5) α-pinene; (6) Borneol; (7) Safrole; (8) Eugenol; (9) D-limonene; (10) Terpinolene

Bray–Curtis PCoA of square-root-transformed relative abundance profiles showed clear separation between distillate and headspace samples, primarily along PCoA1 (sample phase; distillate vs. headspace), which explained 78.9% of the variation (Fig. 1). Mean between-group Bray–Curtis dissimilarity was 0.223, exceeding within-group dissimilarities for both distillate samples (mean = 0.082) and headspace samples (mean = 0.072). Vector fitting identified ten compounds most strongly associated with the ordination configuration: terpinen-4-ol (R² = 0.971), elemicin (R² = 0.966), α-terpineol (R² = 0.959), methyl eugenol (R² = 0.954), α-pinene (R² = 0.875), borneol (R² = 0.784), safrole (R² = 0.776), eugenol (R² = 0.724), limonene (R² = 0.693), and terpinolene (R² = 0.615) (Fig. 1). Consistent with compound-level relative abundance patterns (Table 1), headspace samples were associated primarily with higher relative contributions of α-pinene and limonene, whereas distillate samples were associated with higher relative abundances of elemicin, terpinen-4-ol, α-terpineol, methyl eugenol, and other less volatile constituents.

### Y-Tube Olfactometer Bioassays

**Fig. 2:**
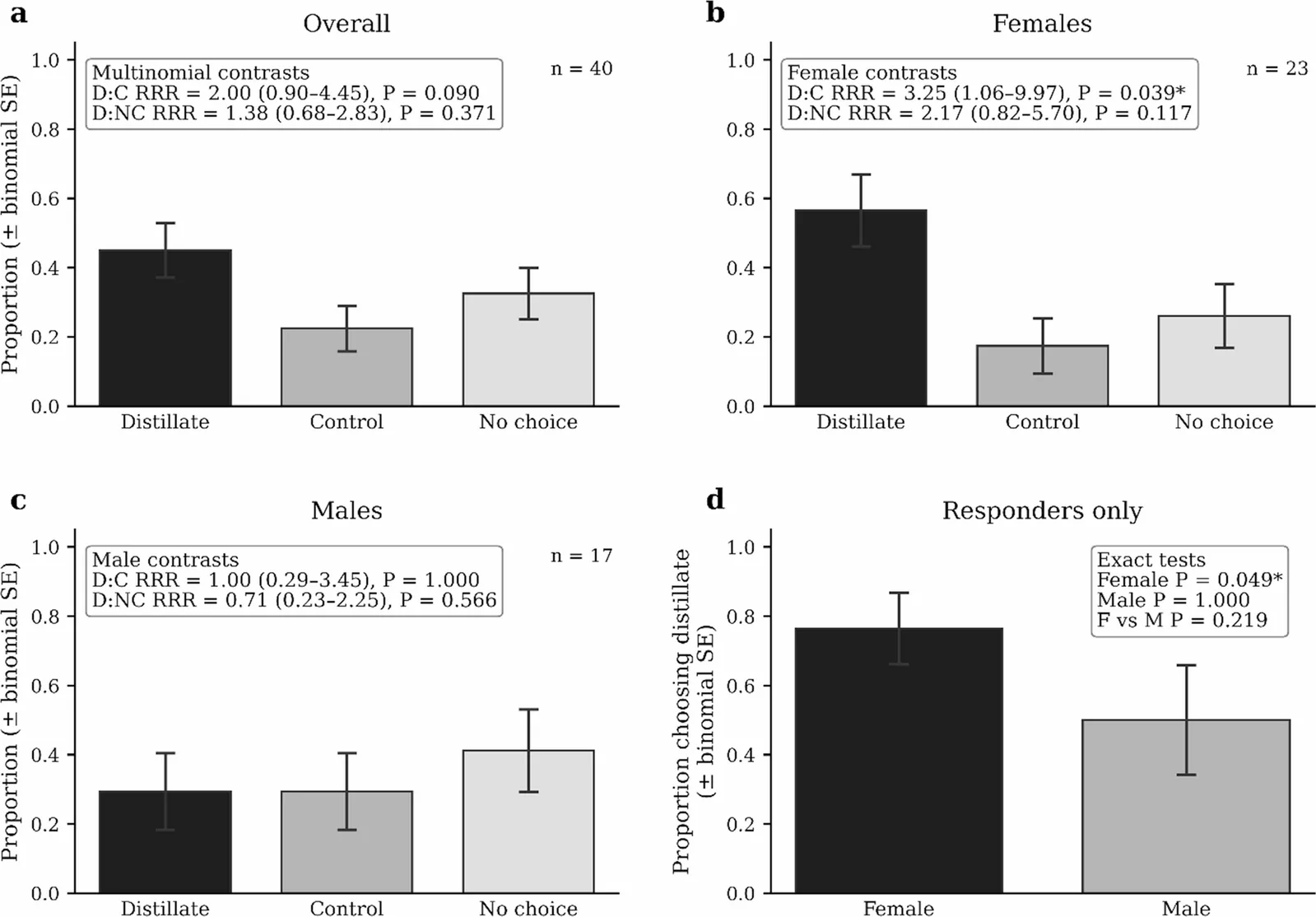
Y-tube olfactometer outcomes for adult *Phloeosinus punctatus* exposed to *Sequoiadendron giganteum* steam distillate. Bars show observed proportions ± binomial SE. Panels show (**a**) all beetles, (**b**) females, (**c**) males, and (**d**) the proportion choosing distillate (**D**) versus control (**C**) among responding females and males. Panels a–c report multinomial relative risk ratio (RRR) contrasts retaining no-choice (NC) responses as a third outcome; panel d reports two-sided exact binomial tests within sex and the two-sided Fisher’s exact female-versus-male comparison

Across all trials (*n* = 40), beetles selected the distillate arm in 18 trials (45.0%), the control arm in 9 trials (22.5%), and made no choice in 13 trials (32.5%) (Table 2; Fig. 2a). Multinomial contrasts retaining no-choice responses as a third outcome indicated that beetles selected the distillate arm more often than the control arm overall, although this contrast did not reach statistical significance (RRR = 2.00, 95% CI 0.90–4.45, *P* = 0.090; Fig. 2a). Females (*n* = 23) selected the distillate arm in 13 trials, the control arm in 4 trials, and made no choice in 6 trials (RRR = 3.25, 95% CI 1.06–9.97, *P* = 0.039; Fig. 2b). Males (*n* = 17) selected the distillate arm in 5 trials, the control arm in 5 trials, and made no choice in 7 trials (RRR = 1.00, 95% CI 0.29–3.45, *P* = 1.000; Fig. 2c).

**Table 2:** Y-tube olfactometer choice outcomes for adult *Phloeosinus punctatus*, overall and by sex, exposed to *Sequoiadendron giganteum* steam distillate versus a control. Values are counts with percentages and Wilson 95% confidence intervals

Group	*n*	Distillate	Control	No choice
Overall	40	18 (45.0% [30.7–60.2])	9 (22.5% [12.3–37.5])	13 (32.5% [20.1–48.0])
Female	23	13 (56.5% [36.8–74.4])	4 (17.4% [7.0–37.1])	6 (26.1% [12.5–46.5])
Male	17	5 (29.4% [13.3–53.1])	5 (29.4% [13.3–53.1])	7 (41.2% [21.6–64.0])

Among responders only (Table 3; Fig. 2d), females exhibited a significant preference for the distillate, with 13/17 responders choosing the distillate arm (76.5%; exact binomial *P* = 0.049). Males did not show a responder-only preference, with 5/10 responders choosing the distillate and 5/10 choosing the control (50.0%; *P* = 1.000). The direct female-versus-male comparison among responders was not statistically significant (sample odds ratio = 3.25, log-Wald 95% CI 0.61–17.28; Fisher’s exact *P* = 0.219).

**Table 3:** Responders-only summary of Y-tube olfactometer choices by *Phloeosinus punctatus* exposed to *Sequoiadendron giganteum* distillate versus control. Distillate-choice percentages are reported with Wilson 95% confidence intervals. Exact binomial tests evaluate distillate preference against H₀: *p* = 0.5

Group	Responders	Distillate	Control	Distillate choice (%)	95% CI	exact binomial *p* (H_0_ = 0.5)
Overall	27	18	9	66.7	47.8–81.4	0.122
Female	17	13	4	76.5	52.7–90.4	0.049
Male	10	5	5	50.0	23.7–76.3	1.000

Exact binomial P-values are reported for tests of distillate versus control against H₀: *p* = 0.5

**Fig. 3:**
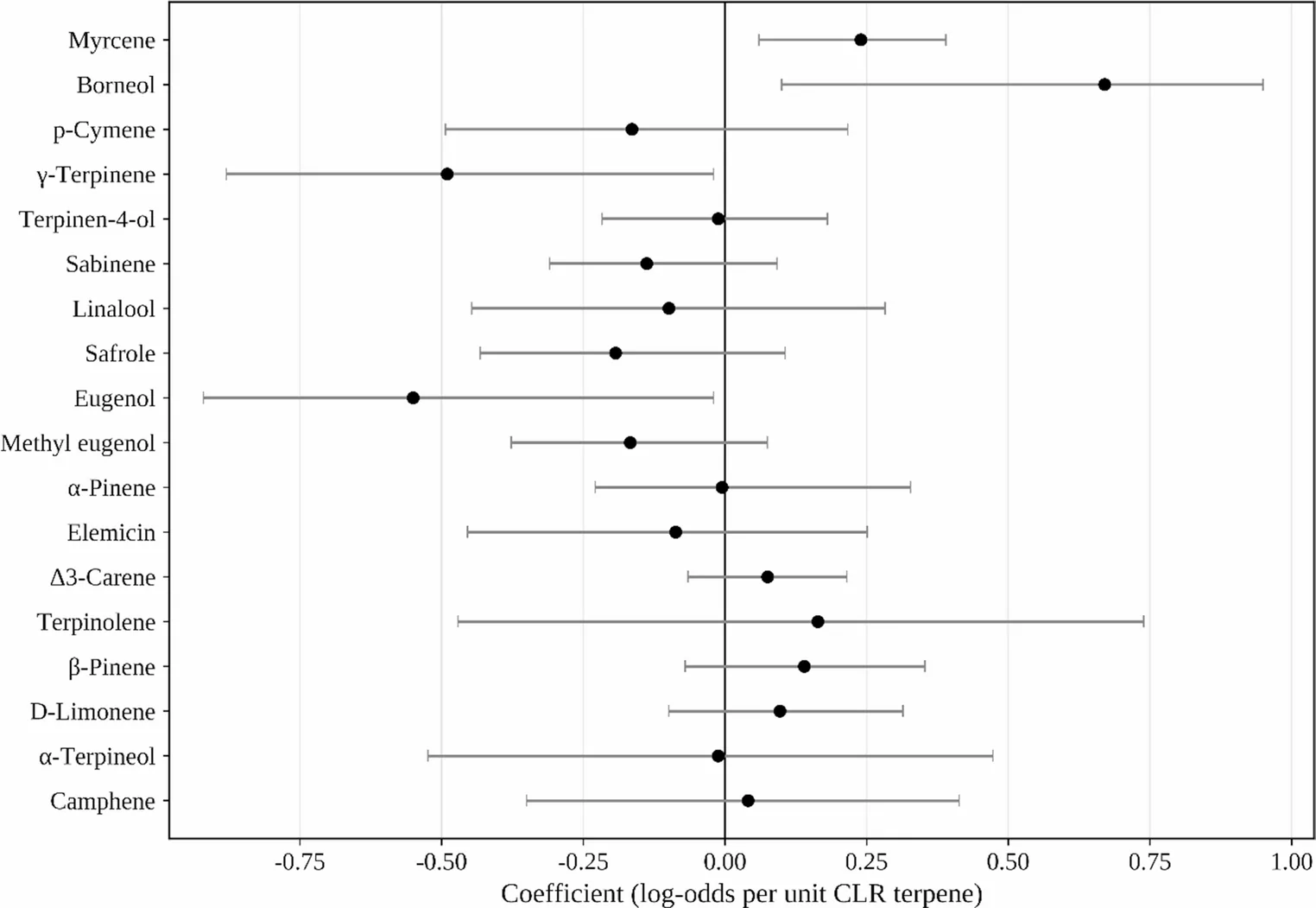
Exploratory associations between giant sequoia distillate headspace composition and female *Phloeosinus punctatus* choice in Y-tube olfactometer assays. Positive coefficients indicate increased odds of choosing the distillate relative to the control, whereas negative coefficients indicate reduced odds. The vertical line at zero denotes no effect. Points represent ridge-penalized logistic-regression coefficients (log-odds per unit centered log-ratio-transformed relative abundance), and horizontal bars are 95% nonparametric bootstrap percentile intervals. Positive and negative coefficients indicate directional covariation with distillate-arm choice across matched profiles from the composite batch; individual compounds were not presented or tested separately

Ridge-penalized logistic regression indicated that borneol (β = 0.67, 95% bootstrap percentile interval 0.10–0.95) and myrcene (β = 0.24, 0.06–0.39) were positively associated with female distillate choice, whereas γ-terpinene (β = −0.49, − 0.88 to − 0.02) and eugenol (β = −0.55, − 0.92 to − 0.02) showed negative associations (Fig. 3). Coefficients for the remaining compounds were less stable across bootstrap resampling, with intervals overlapping zero. Because all constituents were presented as a complete blend and all profiles originated from one composite distillate batch, these coefficients describe mixture-relative, within-batch covariation and do not identify individual compounds as attractants or deterrents.

## Discussion

Giant sequoia distillate elicited a female-biased behavioral response in *P. punctatus* under laboratory conditions. Females selected the distillate arm more often than the control arm, whereas males chose evenly between treatments (Fig. 2; Tables 2 and 3). Although the direct female-versus-male comparison among responders was not statistically significant, the observed pattern is consistent with the hypothesis that females serve as the host-seeking, or “pioneering,” sex in *P. punctatus*, a role that, in monogamous Scolytinae, is associated with locating suitable host or brood material before mate attraction occurs (Vasechko 1978; Kirkendall 1983; Pureswaran and Borden 2003). For example, in the congeneric cypress bark beetle, *P. aubei*, host colonization is initiated by females orienting toward kairomones associated with stress-related shifts in host phytochemistry, after which they produce pheromones that attract mates (Bozsik et al. 2016, 2023, 2026). Several *Ips* species provide equally clear, though directionally opposite, case studies in which males are the pioneer sex. In *Ips typographus* L., males initiate host location and subsequently produce aggregation pheromones (Sun et al. 2006). Likewise, in *Ips paraconfusus* Lanier, host-produced myrcene is converted by males into ipsenol and ipsdienol, which mediate attraction of later-arriving females (Hendry et al. 1980; Seybold et al. 1995).

The lack of preferential orientation by *P. punctatus* males toward the host distillate in our laboratory assays suports the hypothesis that male attraction is relatively dependent on female-produced cues or a mating context not represented in the artificial assay environment utilized here. Importantly, however, our reported olfactometry results do not demonstrate that *P. punctatus* males fail to perceive or use host kairomones under natural conditions. In *P. aubei*, for example, host-derived α-pinene is detected by both sexes (Bozsik et al. 2016, 2022, 2024, 2025). Overall, Y-tube olfactometers constrain insect movement to short-range walking rather than flight, restrict visual cues (e.g., sunlight) present in the insects’ natural habitats, and prevent contact with host material or other natural substrates. Responses may also depend on airflow, odor concentration, release rate, observation period, and beetle condition (Byers 2012; Roberts et al. 2023). Consequently, the apparent sex-biased response observed here should be evaluated using complementary laboratory assays that permit flight or incorporate more realistic odor plumes and substrates (e.g., wind-tunnel, flight-arena, or larger walking-arena assays), followed by field trapping trials under natural host-location conditions.

The distillate headspace was dominated by monoterpene hydrocarbons (Table 1), a compound class broadly characteristic of conifer volatile emissions and repeatedly implicated in scolytine host location, assessment of host vigor, and host volatile–pheromone interactions (Chénier and Philogène 1989; Seybold et al. 2000, 2018; Byers 2004). The primary constituent identified here, α-pinene, is a common host-associated monoterpene and semiochemically active cue for multiple bark beetles, including *Phloeosinus* congeners (Chararas et al. 1980; Werner and Holsten 1984; Miller and Rabaglia 2009). However, because beetles were exposed to the unfractionated distillate, the behavioral responses of *P. punctatus* cannot be attributed to α-pinene or any other individual component of the volatile blend. Bark beetle responses to host odors often depend on blend composition, as constituent monoterpenes can act additively or synergistically to modify attraction (Erbilgin and Raffa 2000; Borden et al. 2008; Zhang et al. 2008). The unfractionated distillate therefore provides a logical starting point for preliminary field trials as a candidate monitoring lure. However, fractionation, antennal screening, and subsequent behavioral testing of individual compounds and reconstructed blends are required to identify which specific constituents of the giant sequoia volatile profile significantly influence orientation behavior in *P. punctatus* (Seybold et al. 2000, 2018; Byers 2004).

Exploratory ridge-penalized logistic regression identified myrcene and borneol as the strongest positive correlates of female distillate choice (Fig. 3). Myrcene is a well-established host volatile and, in other bark beetle lineages, a pheromone precursor (e.g., for ipsdienol) or synergist (Byers and Wood 1979; Seybold et al. 2000, 2018; Byers 2004; Byers and Zhang 2011). Its prominence in the headspace and positive association with female *P. punctatus* choice in this study support its candidacy for future testing as a putative kairomonal component. The positive association of borneol is likewise consistent with studies implicating oxygenated monoterpenes, including bornyl acetate, as semiochemical cues associated with host-tissue oxidation and microbial metabolism of host compounds under stressed-tree conditions (Birgersson and Leufvén 1988; Meurisse et al. 2008; Kandasamy et al. 2023). Several additional compounds present in the distillate but not positively associated with female choice in the penalized logistic regression analysis, including α- and β-pinene, carene, camphene, and limonene, have nonetheless been documented as kairomones for other Scolytinae (Chénier and Philogène 1989; Seybold et al. 2000, 2018; Byers 2004; Bozsik et al. 2026) and therefore warrant consideration in future lure-development work for *P. punctatus*.

The liquid phase of the distillate contained higher relative proportions of less volatile oxygenated terpenes, notably terpinen-4-ol and α-terpineol, and aromatic phenylpropanoids, including safrole, methyl eugenol, and elemicin, than did the airborne headspace (Table 1), producing strong multivariate separation between phases (Fig. 1). Comparable patterns are reported in other conifers, including enrichment of α-terpineol and terpinen-4-ol in *Pinus cembra* L. hydrolates relative to the corresponding distilled oils and oxygenated-monoterpene dominance in *Cupressus sempervirens* L. hydrosol headspace (Paolini et al. 2008; Chizzola et al. 2021; Politi et al. 2022). Because of their lower volatility, these compounds may contribute less to long-range orientation of *P. punctatus*, but they remain plausible candidates for future tests of short-range host acceptance, host suitability assessment, or mate-location behavior (Kandasamy et al. 2023). For example, terpinen-4-ol is a pheromone component in *Polygraphus* Erichson (Rahmani et al. 2019; Viklund et al. 2019) and has been evaluated as a pheromone additive in *Ips* De Geer management (Moliterno et al. 2023; Ramakrishnan et al. 2025). Both terpinen-4-ol and α-terpineol are also produced in the ventriculum of *Phloeosinus armatus* Reitter as possible pheromone constituents (Chararas et al. 1980). Terpinen-4-ol also elicits antennal responses in *P. aubei* (Bozsik et al. 2022), supporting its inclusion in future antennal screening and field-trapping assays for *P. punctatus*.

Among these lower-volatility constituents, the phenylpropanoids that distinguished the distillate from its associated headspace—elemicin, eugenol, and safrole—may be less important for long-range host location but could still influence close-range host acceptance. For instance, ethyl eugenol has been shown to reduce attraction of *Ips grandicollis* Eichhoff to its standard lure, illustrating that aromatic constituents of host volatile profiles can function as disruptants (Afzal et al. 2025). Our analyses and relevant literature suggest γ-terpinene, eugenol, methyl eugenol, and verbenone—an anti-aggregant in *P. aubei*—as useful candidates for future experiments testing chemical deterrence of host-tissue colonization by *P. punctatus* (Fettig et al. 2008; Bozsik et al. 2025, 2026). Overall, field trials comparing whole-distillate formulations, synthetic blends informed by giant sequoia headspace composition, and individual compounds across ecologically relevant doses and release rates will be needed to determine which treatments best elicit attraction or deterrence in *P. punctatus* under natural conditions (Rahmani et al. 2019; Viklund et al. 2019; Kandasamy et al. 2023; Moliterno et al. 2023; Ramakrishnan et al. 2025). These trials should also quantify predator and parasitoid bycatch, as bark beetle natural enemies can detect or orient toward beetle-, host-, and microbe-associated volatiles (Sullivan and Berisford 2004; Hayes et al. 2008; Zhang et al. 2008).

## Conclusions

Our results indicate that giant sequoia distillate contains host-derived volatiles associated with an apparent female-biased orientation response in *P. punctatus* under laboratory conditions. This pattern is consistent with the hypothesis that females serve as the host-seeking, pioneering sex in *P. punctatus*, as occurs in other monogamous scolytines. Replicated field trials should compare the whole distillate with validated candidate compounds and blends across release rates while quantifying predator and parasitoid bycatch. Bioassay-guided fractionation, coupled with GC–EAD or comparable antennal screening, can identify candidate constituents for authentic-standard, dose–response, and reconstructed-blend assays. Together, these efforts could support the development of semiochemical tools for monitoring *P. punctatus* and studying its interactions within giant sequoia–arthropod food webs.

## Data Availability

The datasets supporting the results and analyses reported in this article will be deposited in the Dryad Digital Repository and made publicly available upon publication of the article.
